# Comparing the efficacy of dextrose, mannitol, and saline solutions in male rabbits' chemical castration

**DOI:** 10.1002/ame2.70179

**Published:** 2026-05-12

**Authors:** Mohammad‐Javad Gholizadeh, Reza Azargoun, Ali Shalizar‐Jalali

**Affiliations:** ^1^ Department of Internal Medicine and Clinical Pathology Faculty of Veterinary Medicine, Urmia University Urmia Iran; ^2^ Department of Basic Sciences Faculty of Veterinary Medicine, Urmia University Urmia Iran

**Keywords:** fertility, histopathology, neutering, sterilizing agent, testosterone

## Abstract

Chemical castration offers a noninvasive way to sterilize male animals without surgical complications. An ideal chemical castration agent should be affordable, effective with a single injection, free of side effects, and without negative impacts on animal welfare. Because there is limited research on chemical castration in rabbits, we aimed to investigate the histopathological and hormonal effects of intra‐testicular injections of dextrose, mannitol, and saline solutions in male rabbits. Nine adult rabbits were divided into three equal groups. Under anesthesia, 50% dextrose, 20% mannitol, or 0.9% saline was injected bilaterally into the testicles, with the volume of each solution determined by the width of each testicle. Sixty days after the intra‐testicular injections, the rabbits underwent orchiectomy. All testicular tissues were examined histopathologically. Serum testosterone levels were assessed on days 0 and 60. Histopathological examination showed a significant decrease in the quality and maturity of the seminiferous tubules and spermiogenesis index, as well as an increase in histopathological changes in the testis of the saline‐receiving group compared to other groups (*p* < 0.05). Except for the percentage of mature testicular seminiferous tubules, there was no significant difference between the mannitol‐ and dextrose‐receiving groups (*p* > 0.05). Intra‐testicular injections of any of the solutions did not significantly affect testosterone levels (*p* > 0.05). Our study showed that intra‐testicular injection of saline, unlike dextrose and mannitol, can cause significant testicular tissue damage without side effects. Consequently, 0.9% saline may have potential as a chemical sterilization agent in rabbits.

## Graphical abstract

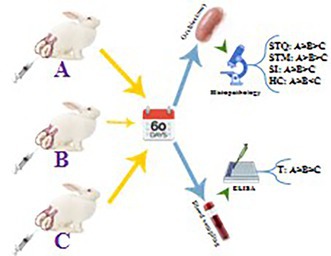

## 1 INTRODUCTION

Neutering small exotic mammals is a common procedure performed by veterinarians.^1^ Male rabbits are often castrated to prevent reproduction, promote social harmony among males, and reduce or eliminate undesirable behaviors, such as urine marking, territorial aggression, or obsessive mating behaviors toward owners, toys, shoes, or other animals.^2^ In recent decades, the use of surgical castration for male neutering has decreased. This decline can be attributed to various factors, such as the high costs associated with surgery, the lengthy procedure, the need for postoperative care, potential surgical complications, and the requirement for anesthesia and sterilization.^3^ Additionally, performing surgical castration in small exotic mammals presents unique challenges, requiring skilled surgeons to have comprehensive knowledge of their anatomy, physiology, and available surgical techniques to ensure successful outcomes.^1^ As a result, alternative methods like chemical castration have gained significant research interest and are considered valuable options.^3^

Chemical castration mainly affects the testis. Various chemical agents injected directly into the testicles have been studied for their ability to cause infertility; many of these are classified as “sclerosing agents.” This group emphasizes the typical tissue responses after intervention, which include necrosis, inflammation, fibrosis, granuloma formation, and blockage of the tubular lumens.^4^ Chemical agents such as chlorhexidine, ethanol, formaldehyde, glycerin, lactic acid, acetic acid, sodium chloride, clove oil, mannitol, atrazine, cetrimide, calcium chloride, zinc compounds, and deslorelin have been tested in different animal models, including donkeys, stallions, buffaloes, wild boars, goats, dogs, cats, ferrets, gerbils, rats, mice, and frogs.^5^ However, few studies have been conducted regarding chemical castration in male rabbits.

Hyperosmotic solutions like dextrose and mannitol can be administered through either continuous or bolus infusions, depending on the goal of treating severe hypoglycemia or reducing increased intracranial pressure, respectively.^6^, ^7^ As an osmotic agent, dextrose may disrupt cellular function and damage membrane integrity through increasing osmotic pressure due to the introduction of a hypertonic solution.^8^ Hypertonic dextrose solution can cause local tissue damage via dehydrating cells at the injection site.^9^ It has been documented that exposure to mannitol causes apoptosis of vascular endothelial cells, resulting in tissue necrosis. Additionally, extravasation of mannitol leads to soft tissue damage through capillary collapse, hypoxia, and necrosis.^7^

Based on the properties of these solutions, it was hypothesized that their intra‐testicular injection, as readily available and inexpensive chemical agents, could induce osmotic shock and cause testicular degeneration. Furthermore, it was assumed that the higher the tonicity of a solution, the more damaging it would be for the testicular tissue. Therefore, this study aimed to compare the potentials of intra‐testicular injection of 50% dextrose, 20% mannitol, and 0.9% saline in the castration of male rabbits.

## 2 MATERIALS AND METHODS

Nine healthy male New Zealand rabbits, aged 1–2 years, with a weight of 1.51 ± 0.38 kg, participated in the study. Before the experiment, the animals underwent a clinical examination. They were housed in suitable cages at the Animal Facility of the Faculty of Veterinary Medicine, Urmia University, Urmia, Iran, for 2 weeks to acclimate to the environment, with unrestricted access to the standard diet and water.

Then, the rabbits were randomly divided into three equal groups, including 50% dextrose (A), 20% mannitol (B), and 0.9% saline (C; control). The volume of the solutions tested was based on the width of each testicle, as described by de Macêdo et al.^10^ Under anesthesia with a combination of xylazine and ketamine (5 mg/kg and 20 mg/kg, IM, respectively), and following surgical preparation of the scrotum, bilateral intra‐testicular injections of chemical agents were performed in each rabbit.^11^ Each injection was given using a sterile 26‐gauge needle, directed from the caudoventral aspect of each testis, approximately 0.5 cm from the tail of the epididymis, toward the dorsocranial aspect.^12^ Before the needle was withdrawn, care was taken to ensure that all the solution had been injected to prevent leakage from the injection site. After completing the injection, the needle was quickly withdrawn, and the testicle was released simultaneously.^13^

Injection into the testes was designated as day 0, and all the rabbits were kept until day 60. During this period, clinical assessments for changes in behavior, food, and water intake, as well as examination of the testicles (i.e., for scrotal swelling, self‐trauma, dermatitis, and scrotal ulcers), were carefully performed once a day for three consecutive days following the injection of the solutions and then once a week until the end of the study. Furthermore, a vernier caliper was used to measure the width of the testicles before and after intra‐testicular injections at the specified time intervals.^14^ Blood samples were collected from the jugular vein of all animals in this study on the morning of days 0 and 60. Blood was kept at room temperature for 45 min to allow coagulation, then centrifuged for 15 min at 3500 rpm, and the serum was stored at −20°C. Serum testosterone levels were assessed using an enzyme‐linked immunosorbent assay kit (Monobind Inc., Lake Forest, USA) following the manufacturer's instructions.^15^

On the final day (day 60), all rabbits underwent bilateral orchiectomy using the pre‐scrotal approach as per the standard method.^16^ Testicular tissues were fixed in 10% buffered formalin. Following conventional tissue processing, the paraffin‐embedded blocks were sectioned to a thickness of 5 μm and stained with hematoxylin and eosin for histopathological examination using light microscopy.^17^, ^18^ The Cosentino scoring system was used to conduct a histopathological examination of testicular tissue. Each testis was assigned a score from 0 to 4 for each parameter (including seminiferous tubules and interstitium), with higher numbers demonstrating a more extensive pathological condition for that parameter.^19^ The Johnsen's scoring method was also used for analyzing spermatogenesis. A total of 100 seminiferous tubules were examined *per* slide, and each slide was scored based on the level of spermatogenesis on a scale of 1–10. Accordingly, a score of 10 indicates maximum spermatogenic activity, whereas a score of 1 indicates the complete absence of germ cells. The percentage of mature seminiferous tubules (scores 9 and 10) was also calculated.^7^ Furthermore, the spermiogenesis index was determined as a percentage of seminiferous tubules with normal spermiation.^20^

The Shapiro–Wilk test was used to assess the normality of the groups' distributions. Statistical analysis was conducted using SPSS version 22.0, and results are presented as mean ± standard deviation. The data were analyzed using one‐way ANOVA, Tukey's post‐hoc test, paired *t*‐test, Kruskal–Wallis test, and Mann–Whitney test. Statistical significance was determined as a *p*‐value of 0.05 or lower.

## 3 RESULTS

Based on clinical assessments, all the studied animals tolerated the intra‐testicular injection of the solutions. None of the rabbits displayed any signs of discomfort or behavioral/nutritional changes throughout the experiment; therefore, the study concluded without any casualties. No adverse effects, including dermatitis, self‐trauma, ulceration, or fistula formation, were identified in the scrotal area after intra‐testicular injections. A temporary, nonsignificant increase in testicular width was noted in all three groups until the second day, which reduced to pre‐injection values after 1 week. Also, no complications related to orchiectomy surgery were observed in the rabbits.

There were no significant differences in the volume of injected solutions (ml) among groups A, B, and C (0.33 ± 0.57, 0.36 ± 0.57, and 0.30 ± 0.05, respectively). Serum testosterone levels pre‐injection (day 0) and post‐injection (day 60) of 50% dextrose, 20% mannitol, and 0.9% saline solutions were 3.35 ± 3.39, 3.30 ± 3.27; 1.38 ± 0.67, 1.70 ± 0.92; and 5.74 ± 3.70, 4.38 ± 2.39, respectively. There was no statistical difference in serum testosterone levels between the different groups. Also, intra‐testicular injections of any of the solutions did not show statistically significant effects on the levels of this hormone.

**FIGURE 1:**
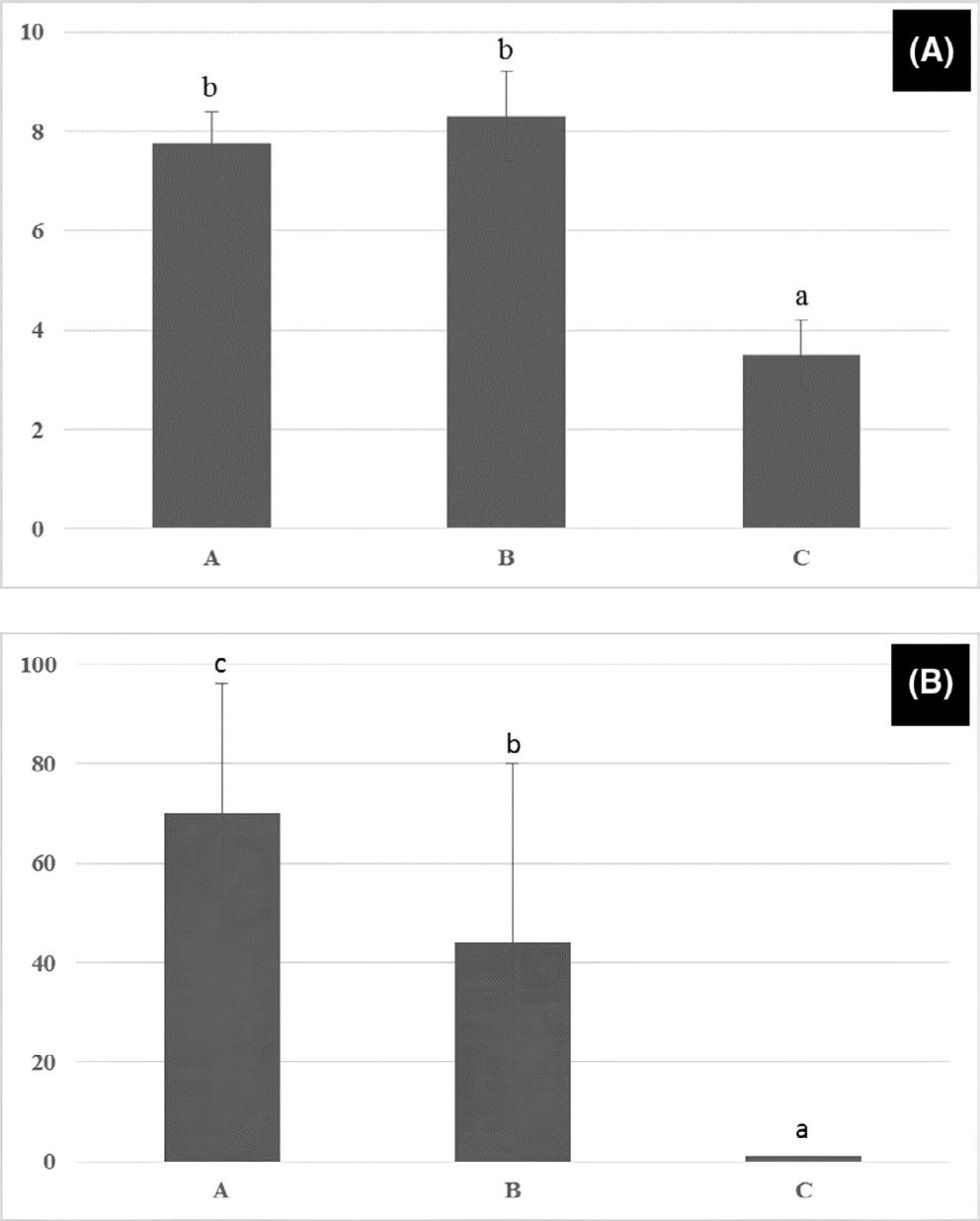
(A) Comparison of testicular biopsy scores based on Johnsen's method. (B) Percentage of mature testicular seminiferous tubules in rabbits that received intra‐testicular injections of dextrose (A), mannitol (B), and saline (C) solutions. Different letters indicate significance at the *p* < 0.05 level.

**FIGURE 2:**
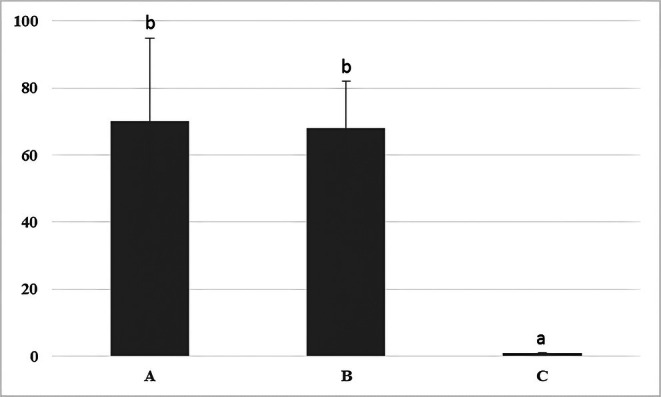
A comparison of spermiogenesis index (%) in rabbits that received intra‐testicular injections of dextrose (A), mannitol (B), and saline (C) solutions. Different letters indicate significance at the *p* < 0.05 level.

Histological evaluations unexpectedly revealed that administration of 0.9% saline caused a significant decrease in the quality and maturity of the testicular seminiferous tubules compared to the other groups (Figure 1). Evaluation of the spermiogenesis index revealed that administration of 0.9% saline resulted in a significant decrease in this index compared to groups A and B (Figure 2).

**FIGURE 3:**
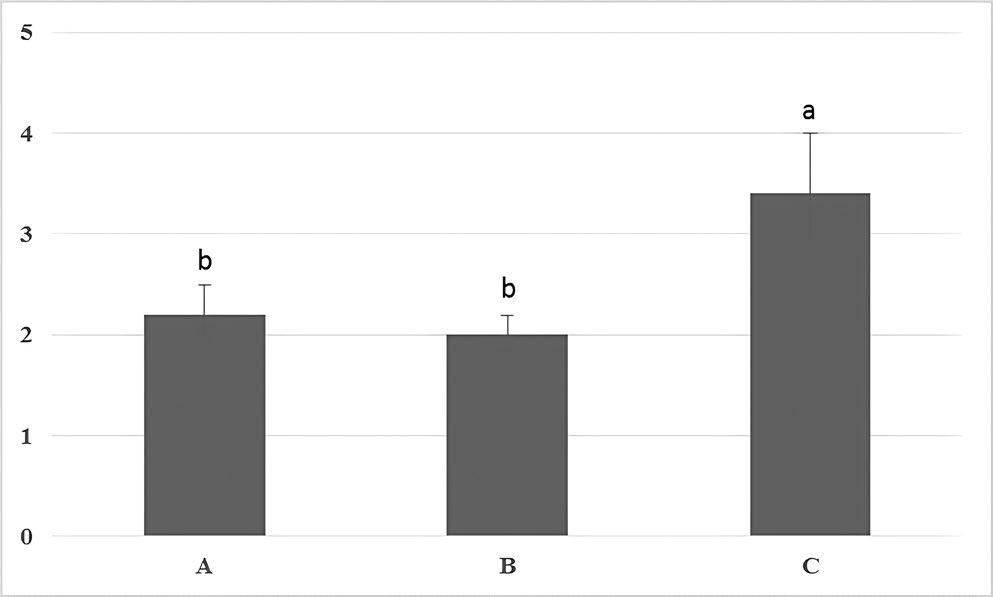
A comparison of testicular histopathological changes based on the Cosentino scoring system in rabbits that received intra‐testicular injections of dextrose (A), mannitol (B), and saline (C) solutions. Different letters indicate significance at the *p* < 0.05 level.

**FIGURE 4:**
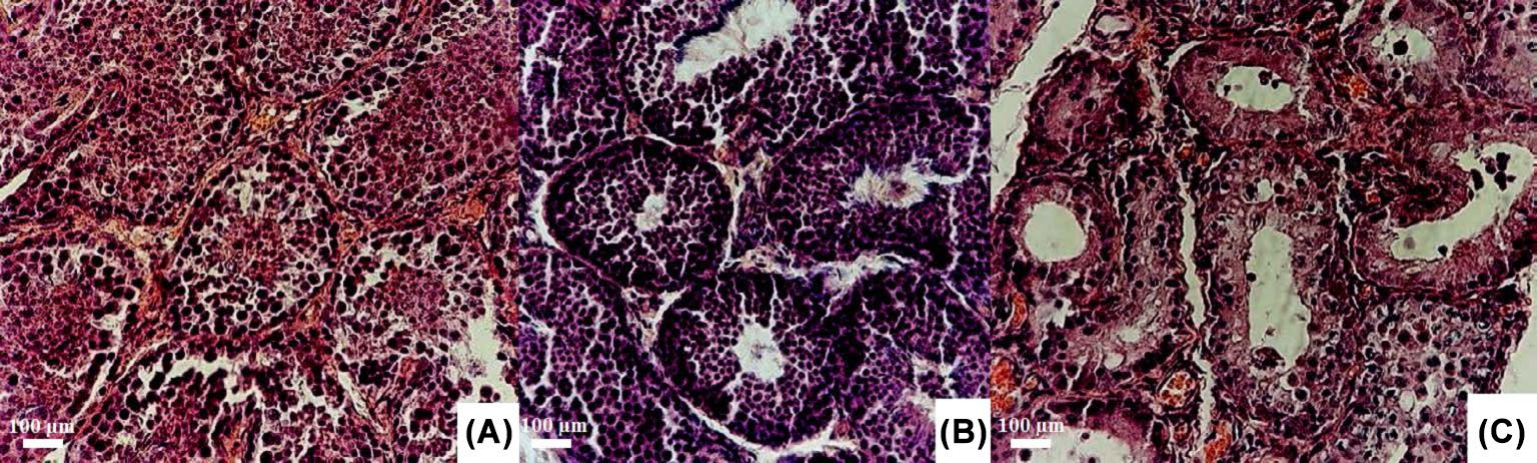
Light micrographic images of cross‐sections of the testis in rabbits that received intra‐testicular injections of dextrose (A), mannitol (B), and saline (C) solutions. In group C, maturation arrest of spermatogenic cells occurs, with spermatogenic cells detaching into the lumen of the seminiferous tubules. In addition, intra‐tubular vacuolization is observed, and there is a complete absence of sperm in the seminiferous tubules of the testis. In contrast, groups A and B do not show significant structural changes in the testicular seminiferous tubules or notable disruption of spermatogenesis. Hematoxylin and eosin staining. Scale bar: 100 µm. Our study demonstrated that a single bilateral intra‐testicular injection of 50% dextrose (Group A) and 20% mannitol (Group B) solutions cannot serve as chemical sterilizing agents in male rabbits. However, intra‐testicular injection of 0.9% saline (Group C) significantly reduced the seminiferous tubule quality (STQ), seminiferous tubule maturity (STM), and spermiogenesis index (SI). In addition, histopathological changes (HC) were more pronounced compared to the other groups. None of the solutions significantly affected testosterone (T) levels.

Histopathological examination showed that intra‐testicular injection of normal saline resulted in a significant increase in histopathological changes in the testis compared to the groups receiving mannitol and dextrose (Figures 3 and 4).

## 4 DISCUSSION

This study examined the hormonal and histopathological effects of a single bilateral intra‐testicular injection of 50% dextrose, 20% mannitol, and 0.9% saline solutions as accessible and affordable chemical agents for sterilizing New Zealand rabbits.

Chemical castration is considered a nonsurgical, bloodless method for neutering male animals.^18^ Various routes, including subcutaneous, intra‐peritoneal, and intra‐venous administrations, have been used to deliver chemical agents to the testes in studies focused on fertility control. However, intra‐testicular injection is the only method that enables the examination of the direct effects of chemicals with unknown toxicity on the testes. Additionally, this method bypasses the systemic metabolism of these agents.^21^ However, research is still ongoing to find a 100% successful, side‐effect‐free approach to end fertility.^3^ Our study did not show any observable side effects in rabbits following intra‐testicular injection of all three solutions. In contrast to our study, significant side effects were observed in dogs following intra‐testicular injections of calcium chloride solutions at concentrations of 30% and 60%. Specifically, 20% of the animals received the 30% solution, and 60% of those given the 60% solution experienced scrotal ulcers, fistulas, and testicular necrosis, necessitating orchiectomy.^22^ The discrepancy may be due to adjusting the volume of solutions for intra‐testicular injection based on the animal's testicular diameter rather than body weight in our study. It has been demonstrated that excessive volumes of chemical agents can cause leakage out of the testicular parenchyma, which may be associated with undesirable side effects.^22^

One key factor in successful castration is lowering testosterone levels to reduce hormone‐driven sexual behaviors. However, in our study, the testosterone level results across all groups were statistically inconclusive. Serum testosterone levels before and after intra‐testicular injection of the solutions in all rabbits remained within the normal range of 0.5–10 ng/mL, as reported by Moor and Younglai.^23^ However, we do not consider this a failure of chemical castration, because testosterone can also be produced by adrenal cortical cells in rabbits.^24^ Excessive production of luteinizing hormone is believed to occur due to reduced negative feedback from the gonads to the hypothalamic–pituitary axis, leading to the secretion of sex hormones from the adrenal glands.^25^ This compensatory mechanism may offset the reduced testosterone production from the testes due to the chemical castration, especially in group C. Furthermore, an ideal castration method should not negatively impact animal welfare.^22^ Research in humans and animals shows that testosterone offers protection against depression, and a deficiency is linked to depression development.^26^ Therefore, although Han et al.^26^ have recommended adjunctive treatment for depression when using both surgical and chemical castrations, our findings suggest that intra‐testicular injection of 0.9% saline in rabbits can result in infertility, confirmed by histopathology, while maintaining normal testosterone levels. It is documented that complete adrenalectomy leads to decreased testosterone levels and reduced sexual behaviors in rabbits.^25^

In the present study, in line with previous research in other animal species,^7^, ^14^, ^27^ 0.9% saline was used for the control group of rabbits; however, the histopathological results were surprising. These findings may result from differences in species' reactions to chemical agents injected into the testicles and/or the doses administered.^28^ In the cat model, Dayanti et al.^27^ found that intra‐testicular injection of normal saline with a volume based on testicular width did not have any adverse effects on testicular parenchyma or spermatogenesis. In contrast, Paranzini et al.^29^ showed that intra‐testicular injection of 0.25 mL of normal saline in cats caused necrosis and degeneration of the seminiferous tubules. In a study by Ali et al.^14^ using a dog model, intra‐testicular injection of normal saline resulted in mild necrotic changes in the testicular parenchyma and Sertoli cells. In a study conducted by Maadi et al.^7^ using a rat model, it was discovered that the intra‐testicular injection of normal saline led to the disruption of the germinal layer. This resulted in the exfoliation of spermatogenic cells into the lumen, which is associated with testicular congestion. Additionally, the normal saline‐administered group showed a significant decrease in the quality and percentage of mature seminiferous tubules.

Regrettably, we did not find any studies exploring the effects of intra‐testicular injection of normal saline in rabbits. However, one probable reason for the histopathological effects observed with 0.9% saline solution in our study may lie in its chemical properties. Although 0.9% saline is commonly referred to as an isotonic or physiological solution, it is neither normal nor physiological compared to the extracellular fluid. This property is believed to be linked to the low sodium‐to‐chloride ratio (1:1) present in 0.9% saline, unlike human extracellular fluid, which has a ratio of 1.38:1, resulting in the occurrence of hyperchloremia.^30^ It is worth noting that the serum sodium and chloride levels in rabbits reflect a similar ratio to those in humans (1.38:1).^31^ The abnormal sodium‐to‐chloride ratio can disrupt ionic homeostasis and affect chloride channels.^30^ Chloride channels are found in nearly every cell in the body, and maintaining appropriate chloride levels is crucial for cellular health. Changes in cellular chloride levels due to dysfunctional chloride channels can lead to various diseases.^32^ Additionally, a 0.9% saline solution has a pH of about 5.4, making it significantly more acidic than the normal pH level of approximately 7.53 in rabbits.^32^ The 0.9% saline solution has also been shown to have toxic effects on the peritoneal cavity, which can be linked to its profibrotic action.^33^

Most importantly, high sodium levels in 0.9% saline can lead to an excessive influx of this cation into cells, driven by the established concentration gradient, which may result in increased intracellular reactive oxygen species (ROS) production.^33^, ^34^ It has been demonstrated that 0.9% saline can induce oxidative stress in various cell types and tissues.^34^ Therefore, it can be assumed that in our experiment, a change in intracellular oxidative stress may also be another probable reason for the occurrence of histopathological changes.

In the rat model, oxidative stress has been shown to cause testicular cyto‐architectural damage. The testicular tissue's sensitivity to oxidation is due to the high levels of polyunsaturated fatty acids and lack of strong protective mechanisms against intense oxidative damage.^3^ Therefore, oxidative stress from injecting 0.9% saline likely caused testicular tissue damage and histopathological changes, including intra‐tubular vacuolization. Intra‐tubular vacuolization can occur due to the degenerative changes or physical exhaustion of germ cells and is recognized as an early morphological indicator of possible Sertoli cell disorders.^3^

Maadi et al.^7^ have demonstrated that the intra‐testicular injection of 20% mannitol and 20% sodium chloride can effectively induce infertility in rats. These authors believed that degenerative changes in the testicular tissue occurred due to the severe osmotic shock and cellular dehydration following injection of these solutions. Accordingly, it was conceivable that intra‐testicular injection of 50% dextrose, due to its hypertonic nature, would similarly lead to infertility. However, our results indicated that this solution was almost ineffective on rabbit testicular tissue. The effects of 20% mannitol on the testicular tissue of the rabbits in this study may be linked to increased levels of ROS. A recent study by Baqerkhani and colleagues showed that intra‐testicular injection of 20% mannitol in donkeys can induce oxidative stress, leading to testicular tissue destruction and ultimately infertility.^3^ In our study, the milder effects observed from intra‐testicular injection of 20% mannitol may be attributed to species differences compared to the findings in two previous studies. For example, although hypertonic saline in rats was found to be a chemical sterilizing agent, a comparable study in adult dogs showed the preservation of androgenesis.^35^

This study had two primary limitations that should be acknowledged. One was the small number of animals studied due to insufficient facilities, which limited statistical power. Another was the lack of semen analysis and mating trials, which were not the purpose of this study but could have confirmed infertility.

In conclusion, our study showed that neither 50% dextrose nor 20% mannitol can be used as chemical sterilizing agents in a male rabbit model. However, a single bilateral intra‐testicular injection of 0.9% saline, which caused significant testicular tissue damage without adverse effects, could potentially replace surgical castration in New Zealand rabbits. The observed histopathological changes could possibly be associated with oxidative stress induced by 0.9% saline or its chemical properties. Therefore, it is advised not to use this solution as a solvent or diluent for chemical agents in studies on rabbit sterilization. It is important to note that for species‐specific use of 0.9% saline as a sterilizing agent, these findings should be supported by longer‐term studies.

## AUTHOR CONTRIBUTIONS

**Mohammad‐Javad Gholizadeh:** Conceptualization; data curation; formal analysis. **Reza Azargoun:** Investigation; methodology; project administration; writing – original draft. **Ali Shalizar‐Jalali:** Resources; software; supervision; validation.

## FUNDING INFORMATION

The authors thank the Research Vice‐Chancellor of Urmia University for the financial support of this project.

## CONFLICT OF INTEREST STATEMENT

None to declare.

## ETHICS STATEMENT

All procedures in the current study were approved by the Animal Ethics Committee of Urmia University, Urmia, Iran (Ref: IR‐UU‐AEC‐3/59).

## Data Availability

All data are available in the main text.
